# A Review of Malaysian Herbal Plants and Their Active Constituents with Potential Therapeutic Applications in Sepsis

**DOI:** 10.1155/2020/8257817

**Published:** 2020-10-28

**Authors:** Kong Yen Liew, Md Faizul Hafiz, Yi Joong Chong, Hanis Hazeera Harith, Daud Ahmad Israf, Chau Ling Tham

**Affiliations:** Department of Biomedical Sciences, Faculty of Medicine and Health Sciences, Universiti Putra Malaysia, 43400 UPM Serdang, Selangor Darul Ehsan, Malaysia

## Abstract

Sepsis refers to organ failure due to uncontrolled body immune responses towards infection. The systemic inflammatory response triggered by pathogen-associated molecular patterns (PAMPs), such as lipopolysaccharide (LPS) from Gram-negative bacteria, is accompanied by the release of various proinflammatory mediators that can lead to organ damage. The progression to septic shock is even more life-threatening due to hypotension. Thus, sepsis is a leading cause of death and morbidity globally. However, current therapies are mainly symptomatic treatment and rely on the use of antibiotics. The lack of a specific treatment demands exploration of new drugs. Malaysian herbal plants have a long history of usage for medicinal purposes. A total of 64 Malaysian plants commonly used in the herbal industry have been published in Malaysian Herbal Monograph 2015 and Globinmed website (http://www.globinmed.com/). An extensive bibliographic search in databases such as PubMed, ScienceDirect, and Scopus revealed that seven of these plants have antisepsis properties, as evidenced by the therapeutic effect of their extracts or isolated compounds against sepsis-associated inflammatory responses or conditions in *in vitro* or/and *in vivo* studies. These include *Andrographis paniculata*, *Zingiber officinale*, *Curcuma longa*, *Piper nigrum*, *Syzygium aromaticum*, *Momordica charantia*, and *Centella asiatica*. Among these, *Z. officinale* is the most widely studied plant and seems to have the highest potential for future therapeutic applications in sepsis. Although both extracts as well as active constituents from these herbal plants have demonstrated potential antisepsis activity, the activity might be primarily contributed by the active constituent(s) from each of these plants, which are andrographolide (*A. paniculata*), 6-gingerol and zingerone (*Z. officinale*), curcumin (*C. longa*), piperine and pellitorine (*P. nigrum*), biflorin (*S. aromaticum*), and asiaticoside, asiatic acid, and madecassoside (*C. asiatica*). These active constituents have shown great antisepsis effects, and further investigations into their clinical therapeutic potential may be worthwhile.

## 1. Introduction

The term sepsis was historically used to describe rotting flesh or putrefaction [1]. In the current medical context, sepsis refers to a condition in which dysregulated immune responses towards infection lead to organ failure [2]. Sepsis may develop secondarily to bacterial, viral, fungal, or parasitic infections [3]. At present, the terms “sepsis” and “severe sepsis” are used interchangeably, whereas “septic shock” refers to a subset of sepsis which is associated with circulatory and metabolic abnormalities and thus increased risk of mortality [4].

Sepsis can be triggered by infectious agents or certain molecules they express, also known as pathogen-associated molecular patterns (PAMPs) [3]. The pathogens or PAMPs may cause direct toxicity and injury to tissues, but in the context of sepsis, the underlying damage is primarily attributed to the immune responses [5]. The exposure of immune cells to PAMPs, such as lipopolysaccharide (LPS), will trigger numerous responses in the body, including vasodilation to increase recruitment of immune cells, followed by rapid production of cytokines by activated immune cells to eradicate the invading pathogens [6]. While such cellular responses are required to remove the pathogens, the same responses could also lead to profound disturbance and harm to the host. Vasodilation may subsequently lead to shock, whereas the cytokine storm may cause tissue damage and eventually leads to organ dysfunction and failure [7]. It is, in essence, a form of “friendly fire” that arises from the body's attempt to eradicate the pathogens.

Despite the long history of sepsis, a significant improvement in its morbidity and mortality rate is yet to be observed. Sepsis was estimated to affect 30 million people worldwide, with up to 8 million fatalities each year [8]. In fact, the number of fatalities is expected to be greater since the epidemiological data on sepsis incidence and mortality rates from low- to middle-income countries was scarce [9]. In the United States alone, an estimated 1.7 million of sepsis cases occur among adults, resulting in more than 250 000 deaths each year [10]. Despite advancement in the healthcare system, the incidence of sepsis and associated mortality in the United States hospitals remained high from 2009 to 2014 [11]. The disease burden of sepsis is immense due to the enormous amount of healthcare costs spent [8].

In Malaysia, sepsis is a major cause of morbidity and mortality, particularly among ICU patients. In 2016 and 2017, sepsis was the most common diagnosis leading to ICU admission. Furthermore, in-hospital mortality rate following ICU admission due to sepsis also recorded an astonishing figure—more than 50% for six consecutive years since 2012 [12]. An eight-year review of blood culture from sepsis cases in the Emergency Department in a Malaysia hospital revealed that 55.2% of the isolated organisms were Gram-negative bacteria. The bacteria were also found to be more resistant to antibiotics commonly prescribed in the outpatient setting [13]. These findings highlight an urgent need to reduce the burden caused by sepsis both in Malaysia and worldwide.

## 2. Molecular Mechanisms in the Pathogenesis of Sepsis

Sepsis could be caused by bacterial, viral, fungal, or parasitic infections [3]. It is initiated when the initial host response to an infection becomes amplified and dysregulated, resulting in the loss of the balance between proinflammatory and anti-inflammatory responses [14, 15]. The recognition of PAMPs, such as bacterial endotoxins and fungal *β*-glucans, by pathogen recognition receptors (PRRs) expressed by the immune cells is considered the first event that triggers the innate immune responses [16, 17]. Innate immunity reacts immediately to the invading pathogens and thus plays a major role in the initiation of sepsis [15]. The binding of LPS, a well-recognized PAMP on the outer cell wall of Gram-negative bacteria, to toll-like receptor 4 (TLR4) will activate intracellular signaling pathways that promote the production of inflammatory mediators such as cytokines, chemokines, and nitric oxide (NO) [7, 16]. This is mainly mediated by the transcription factor nuclear factor-*κ*B (NF-*κ*B) that plays a crucial role in modulating inflammation by activating the transcription of various proinflammatory cytokines [16].

The vast array of inflammatory cytokines released during sepsis include proinflammatory cytokines, such as interleukin (IL)-1, IL-6, IL-12, tissue necrosis factor (TNF)-*α*, interferon (IFN)-*γ*, and macrophage migration inhibitory factor (MIF) and anti-inflammatory cytokines, such as IL-10, IL-14, and transforming growth factor (TGF)-*β*. The uncontrolled release of these cytokines, which can damage tissue and organ by triggering inflammatory cascade, presents a major threat to sepsis patients [7]. Another important proinflammatory cytokine—high mobility group box (HMGB)-1, is a late phase mediator of sepsis. Its production is regulated by NF-*κ*B activation, but the exact mechanism still remains unclear [18]. HMGB-1 has pleiotropic effects on different immune cells, triggering the release of proinflammatory cytokines from peripheral blood mononuclear cells and endothelial cells, as well as inducing the maturation of dendritic cells [19]. Besides that, sepsis is also linked to prostaglandins (PGs), which are lipid mediators produced by cyclooxygenase (COX) enzyme in response to inflammation or infection [20]. Since PGs are involved in various unfavorable outcomes of sepsis, such as impaired tissue perfusion and organ failure, COX inhibitors targeting the inducible form of COX (COX-2) had been tested clinically, but none of the studies reported a promising outcome [21].

Apart from causing organ damage, inflammatory mediators could also cause injury on the endothelial lining of the blood vessels, leading to endothelial dysfunction—a common feature of both sepsis and septic shock [16]. Endothelial dysfunction is accompanied by increased permeability, which could lead to vascular leakage and subsequently life-threatening conditions due to edema and hypotension [22]. Under normal physiological condition, NO is produced by endothelial cells using the constitutively expressed form of the nitric oxide synthase (NOS) enzyme. However, excessive production of NO by the inducible form of NOS (iNOS) was found to contribute to hypotension and vascular abnormalities in patients with septic shock [23]. Damaged endothelial cells also release tissue factors that may activate a coagulation cascade which in turn could disturb the homeostatic balance in sepsis patients [17].

Collectively, the processes described above indicate the complex nature of sepsis which involves the interaction between different types of immune responses and mediators. This complexity that underlies sepsis may partly explain why the development of an effective treatment for sepsis is challenging.

## 3. An Unmet Need for Sepsis-Specific Treatment

Despite the attention and research interest sepsis has received, FDA-approved drugs to treat sepsis are still lacking. The current guideline for the treatment of sepsis is centered loosely on the Surviving Sepsis Campaign. The campaign was launched in 2004 [24] and updated every four years [2, 25, 26], including a revised bundle developed as an update to the 2016 edition [27]. The campaign defined numerous treatment procedures and methods to deal with sepsis patients, which mainly emphasize initial resuscitation, early diagnosis, and antimicrobial therapy [2].

The Surviving Sepsis Campaign had yielded positive outcome in the survival rate of sepsis patients; however, the mortality rate remained high at 27.2% and 36.7% for ICU and in-hospital patients, respectively [28]. One possible reason could be the lack of a sepsis-specific treatment that targets the hyperinflammatory state of the patients. As described earlier, sepsis is a hyperinflammatory disease, with pathogen as the causative agent. While eradication of the pathogen using antimicrobial therapy may be effective in reducing the mortality rate of sepsis, a combined therapy with anti-inflammatory agents may further improve the survival rate of sepsis patients. Nevertheless, another challenge to pursue this alternative is the fact that anti-inflammatory drug specific for sepsis is lacking.

In order to develop drugs for the treatment of sepsis, several strategies which specifically target sepsis-associated cytokines, inflammatory enzymes, or the coagulation cascade have been attempted. These include TNF-*α* inhibitor [29], TNF receptor p55 inhibitor [30], IL-1 inhibitor [31], anticoagulant therapy [32], and nitric oxide synthase inhibitor [33], but none of these enhanced the survival rate of sepsis patients. Nevertheless, a recombinant form of human activated protein C, drotrecogin alfa (activated), was approved by FDA in 2001 for the treatment of severe sepsis after it was demonstrated to improve the patients' survival rate in the PROWESS trial [34]. However, drotrecogin alfa (activated) was later withdrawn from the market in 2011 after the follow-up PROWESS SHOCK trial failed to replicate the promising results reported earlier [35]. On the contrary, the advantage of using corticosteroids remains elusive as a recent meta-analysis revealed that the use of corticosteroids in critically ill patients with sepsis possibly results in a small reduction in mortality but, at the same time, also possibly increases the risk of neuromuscular weakness [36]. Two other drugs, thrombomodulin alfa (ART-123) and toraymyxin, are currently in phase III clinical trials, while several others are yet to reach phase III [37].

Prior to entering clinical trials, potential therapeutic agents for sepsis would be evaluated for their efficacy in the preclinical studies using different experimental models. Sepsis animal models are generally divided into three categories: endotoxemia model (e.g., LPS infusion), bacterial infection model (e.g., *Escherichia coli* infusion), and host-barrier disruption model (e.g., cecal ligation and puncture (CLP) and colon ascendens stent peritonitis (CASP)) [38]. For LPS-induced endotoxemia, the animals are occasionally presensitized with D-galactosamine to reduce the dose of LPS required to generate an inflammatory response [39]. CLP-induced polymicrobial sepsis is currently the most widely used model due to its close resemblance to the progression and clinical characteristics of human sepsis [40]. Despite showing efficacy in the preclinical studies, many therapies have failed to produce a promising outcome in the clinical settings [38].

Taken together, the lack of a specific antisepsis treatment is still an unresolved issue and thus highlights the need to prioritize the exploration of new drugs. This is particularly important as the continuous use of antibiotic therapy to treat sepsis may promote antibiotic resistance which will be another great challenge for healthcare professionals.

## 4. Insights into Malaysian Herbal Plants

Given the unsuccessful attempts on the development of drugs for sepsis treatment so far, it may be worthwhile to explore other alternatives such as herbal-based medicine. Herbal-based medicine has increasingly gained attention since Professor Tu Youyou was awarded a Nobel Prize in Physiology or Medicine in 2015 for her discovery of artemisinin as a treatment for malaria. Artemisinin was actually discovered through the screening of herbs based on ancient manuscripts [41]. Thus, it is highly encouraging that herbal-based medicine may have the potential to be further developed for the treatment of sepsis.

Malaysia is a country rich in flora and fauna, with diverse culture and practices influenced mainly by Malay, Chinese, and Indian backgrounds. Hence, various plants in Malaysia have been used for medicinal purposes based on different cultures and practices [42]. This review aims to provide detailed information of some Malaysian herbs that may have the potential to treat sepsis.

## 5. Methodology

A total of 64 herbal plants were documented in Malaysian Herbal Monograph 2015 (See Supplementary Material-Table A), which extensively describes their traditional uses and proven pharmacological activities. Such information is also available online in the Globinmed website (http://www.globinmed.com/), created by Institute for Medical Research under Ministry of Health Malaysia. A bibliographic search was performed in the following databases: PubMed, ScienceDirect, and Scopus, to find any sepsis-related study on each of the 64 herbal plants listed in Malaysian Herbal Monograph 2015. The search terms used were (“scientific name of the plant” or “common name of the plant”) and (“sepsis” or “lipopolysaccharide” or “LPS”) without narrowing or limiting search items, and the last search was performed on 4 March 2020. All publications obtained from the databases with the searching criteria were studied. Herbal plants that have not been tested, either in the form of extracts or compounds isolated from the plants, in any sepsis-related study were excluded.  Table 1 summarizes seven Malaysian herbal plants that have been studied for antisepsis properties, evidenced by the therapeutic effect of the extracts or compounds extracted from these plants against sepsis-associated inflammatory responses or conditions in *in vitro* or/and *in vivo* experimental models. These mainly include LPS- and LPS-/IFN-*γ*-induced macrophages (*in vitro*) and *E. coli*-infected mice, LPS-induced endotoxemia, and CLP-induced polymicrobial sepsis (*in vivo*). Besides that, *in vitro* or *in vivo* studies that used HMGB-1 or Transforming Growth Factor-Beta-Induced Protein (TGFBIp) as an inducer to induce endothelial barrier dysfunction were also included. This is because endothelial dysfunction plays an important role in the pathogenesis of sepsis leading to multiple organ failure [88], and thus, therapeutic agents that effectively inhibit endothelial barrier dysfunction may have beneficial effect in sepsis. It should be noted that active constituents which only demonstrated *in vitro* pharmacological effects and lack supporting *in vivo* studies were not included in Table 1 but were briefly discussed under their respective herbal plants below. The anti-inflammatory effects of these active constituents as evidenced by the *in vitro* findings alone are not sufficient to support their antisepsis potential.

## 6. Malaysian Herbal Plants and Their Active Constituents with Potential Therapeutic Applications in Sepsis

### 6.1. *Andrographis paniculata*

*Andrographis paniculata* (*A. paniculata*), vernacularly known as King of Bitters, is a bushy medicinal plant distributed extensively in Southeast Asia and Southern Asia. In Malaysia, the plant is called *“hempedu bumi”* or *“pokok cerita”* and commonly found at the roadsides, on the hills, and in the forest bed. Traditionally, it has been used for cold, malaria, snake bites, diabetes, and hypertension [89]. Some of the effects of *A. paniculata* that have been scientifically proven include its antibacterial, antioxidant, antidiabetic, and anti-inflammatory effects [90–92].

The ethyl acetate extract of *A. paniculata* was previously found to have anti-inflammatory effects on LPS-/IFN-*γ*-induced macrophages and LPS-treated mice [43, 44]. An oral administration of the ethyl acetate extract (0.78–1.32 mg/kg) improved the survival rate of endotoxemic mice and reduced the serum levels of NO, TNF-*α*, and macrophage inflammatory protein (MIP)-2, all of which play proinflammatory roles in sepsis [44]. A bioassay-guided fractionation study revealed that eight bioactive compounds contributed to the inhibition of NF-*κ*B transcriptional activity of the ethyl acetate fraction, with the two most abundant compounds in the fraction being andrographolide and 14-deoxy-11,12-didehydroandrographolide [93]. In particular, andrographolide has been shown to exhibit barrier protective effect in HMGB-1-induced endothelial cells by decreasing permeability and monocytes adhesion and transmigration. Furthermore, andrographolide administered via intravenous injection was able to reduce sepsis-induced HMGB-1 production, inhibit vascular leakage, and improve the survival rate of CLP-treated mice [45]. Other studies have also reported similar *in vivo* protective effect where intraperitoneal injection of andrographolide attenuated acute lung injury (ALI) and acute liver injury in LPS-induced and LPS-/D-galactosamine-induced mice, respectively [46, 47]. Collectively, these findings suggest that andrographolide possesses strong antisepsis potential.

Apart from andrographolide, several studies have demonstrated that other bioactive compounds isolated from *A. paniculata* also possess anti-inflammatory activity. For example, isoandrographolide and neoandrographolide are two andrographolide derivatives which have been reported to have the capability to inhibit the production of proinflammatory mediators, such as NO, PGE_2_, IL-1*β*, IL-6, and TNF-*α*, in LPS-induced macrophages [94–96]. Two other bioactive compounds, namely, skullcapflavone-1 and 7-O-methylwogonin, were also reported to exert *in vitro* inhibitory effect against LPS-induced NO and PGE_2_ production in macrophages [96]. Besides that, andrograpanin which was reported by Liu et al. [97] to inhibit the production of inflammatory mediators in LPS-induced macrophages and other bioactive compounds which were reported by Chao et al. [93] to inhibit NF-*κ*B transcriptional activity are other active constituents from *A. paniculata* that should be further studied for their antisepsis potential.

### 6.2. *Zingiber officinale*

*Zingiber officinale* (*Z. officinale*), or ginger, belongs to the Zingiberaceae family. The ginger rhizome has a long history of medicinal use among Chinese and Indian community. It is now widely cultivated in various tropical countries, especially India. There are also wild varieties of this plant available in tropical and subtropical countries, including Malaysia. The health benefits of ginger are numerous, including antioxidant, anti-inflammatory, anticarcinogenic, and prevention of cardiovascular diseases [98–101].

The anti-inflammatory activity of ginger is supported by a study which reported that oral administration of dried ginger dissolved in distilled water was able to inhibit LPS-induced inflammation in a mouse model by suppressing the production of IL-6 and IFN-*γ*, mainly through the inhibition of NF-*κ*B signaling pathway [52]. Furthermore, the same study also demonstrated that the ginger extract could ameliorate liver damage, most likely due to its ability to reduce the expression of inflammatory enzymes (iNOS and COX-2) in the liver [52]. This is consistent with another study which reported that the inhibition of iNOS and COX-2 was able to abrogate liver injury [102]. These results suggest that the ginger extract may be beneficial not only as an anti-inflammatory agent but also to protect against tissue and organ damage commonly observed in severe sepsis cases.

An *in vitro* study by Tripathi et al. [48] demonstrated that the alcoholic extract of ginger suppressed LPS-induced activation of macrophages as indicated by decreased production of proinflammatory cytokines (IL-1*β*, IL-12, and TNF-*α*) and chemokines (Regulated on Activation Normal T-Cells Expressed and Secreted (RANTES) and monocyte chemoattractant protein (MCP)-1). Another study also reported that the major constituent of *Z. officinale*—6-gingerol—exhibited similar inhibitory effects on LPS-stimulated macrophages [49], suggesting that the anti-inflammatory effects of the alcoholic extract of ginger reported previously [48] were largely attributed to 6-gingerol. Moreover, 6-gingerol was also shown to decrease the serum levels of alanine aminotransferase (ALT) and aspartate aminotransferase (AST), two common biomarkers of liver injury, in CLP-induced polymicrobial sepsis [53]. Indeed, both its anti-inflammatory activity and its ability to suppress liver injury further highlight the antisepsis potential of 6-gingerol.

Other studies have also investigated the activity of zingerone, a phenolic alkanone found in *Z. officinale*, using *in vitro* and *in vivo* models. In particular, zingerone has been shown to inhibit the production of HMGB-1 and TGFBIp in LPS-induced human umbilical vein endothelial cells (HUVECs), as well as in CLP-induced septic mice [50, 51]. Both HMGB-1 and TGFBIp are proinflammatory mediators that promote vascular leakage in sepsis [19, 51]. Zingerone was able to inhibit HMGB-1- and TGFBIp-induced endothelial dysfunction through the suppression of hyperpermeability, cell adhesion molecules (CAMs) expression, and adhesion and transendothelial migration of neutrophils [50, 51]. Most importantly, zingerone's therapeutic effect is evidenced by its ability to decrease sepsis-related mortality and to reverse organ damage such as hepatic, renal, and pulmonary injury in CLP-induced septic mice at a dose of 0.72 mg/kg via intravenous injection [50]. Similar improvements in the survival rate and tissue damage were also observed in TGFBIp-induced CLP mice injected with zingerone (0.36 and 0.72 mg/kg) intravenously [51]. These findings are in line with other reports that described the ability of zingerone to alleviate lung and kidney injury in LPS-induced mice upon intragastric and intraperitoneal administration, respectively [54, 55]. These studies cumulatively suggest that *Z. officinale* or ginger has great therapeutic potential for sepsis by suppressing sepsis-related mediators and organ damage.

Other than 6-gingerol and zingerone, there are various active compounds isolated from ginger that have also shown great anti-inflammatory activity in LPS-induced macrophages, especially the shogaols, gingerols, and gingerdiones. Among the shogaols, 6-shogaol has been reported by a few *in vitro* studies to suppress LPS-induced inflammatory mediator production by inhibiting the expression of iNOS and COX-2 [103–105]. Moreover, 1-dehydro-10-gingerdione has also been shown to have similar inhibitory activity on iNOS expression, resulting in reduced production of NO [103]. Other gingerols (8- and 10-gingerol) and gingerdiones (1-dehydro-6-gingerdione and 12-dehydrogingerdione) are just a few among those that have been demonstrated to have *in vitro* anti-inflammatory activity [105–108] and may be further investigated *in vivo* for their potential therapeutic use for sepsis.

### 6.3. *Curcuma longa*

*Curcuma longa* (*C. longa*), usually known as turmeric, has been used for thousands of years in the Ayurvedic tradition as well as for culinary purpose. The plant, which belongs to the Zingiberaceae family, is a perennial herb known as “*kunyit*” in Malaysia. In folk medicine, *C. longa* is frequently used for the management of asthma, gonorrhea, helminthic diseases, and urinary tract disorders [109]. Rhizome of *C. longa* is known to possess several therapeutic properties such as anti-inflammatory, antioxidant, antibacterial, antiviral, antifungal, and antidiabetic properties [110–114].

A previous study reported that oral administration of *C. longa* extract-loaded nanoemulsion (CLEN) was able to improve the survival rate of LPS-induced lethal endotoxemic mice [56], indicating the antisepsis potential of *C. longa*. The protective effect of CLEN against endotoxin-induced mortality was associated with its ability to reduce serum levels of HMGB-1 and the expression of iNOS in various tissues including heart, lung, liver, and kidney [56].

Curcumin is a major compound found in the rhizome of *C. longa* and has been extensively studied for its antisepsis potential. Intravenous injection of curcumin rescued mice from CLP-induced lethality, lowered the levels of tissue injury markers, such as ALT, AST and lactate, and inhibited the production of TNF-*α* [57]. Intraperitoneal injection of curcumin also improved the survival rate and attenuated blood-brain barrier dysfunction in CLP-induced mice [58]. Apart from that, curcumin, administered via intranasal, intraperitoneal, and oral routes, exerted protective effects against organ dysfunction in LPS-induced endotoxemia, including acute lung injury and liver injury [59–61]. Notably, a more recent study by Kumari et al. [62] found that intraperitoneal, but not intranasal, administration increased the survival of LPS-induced mice and decreased TNF-*α* production. The failure of curcumin to yield reproducible results via intranasal administration suggests that it might not be an appropriate route of administration for curcumin because there are many factors that affect the bioavailability of intranasally administered drugs including their volume and concentration, and the absorptive surface of nasal mucosa [115] and thus an optimal formulation should be determined beforehand. Last but not least, curcumin also demonstrated beneficial effects on the cardiovascular system as intragastric injection of curcumin has been shown to prevent myocardial injury in CLP-induced septic mice [63], while intraperitoneal injection of curcumin has been shown to improve vascular function in LPS-challenged mice by maintaining a normal heart rate and blood pressure [64]. Based on these reports, it is evident that curcumin may prevent organ damage due to sepsis and thus have promising potential for the treatment of sepsis.

Some studies have also identified other active compounds from *C. longa* with anti-inflammatory activity. Some examples include turmeronol A and B, (6S)-2-methyl-5-hydroxy-6-(3-hydroxy-4-methylphenyl)-2-heptene-4-one, (6S)-2-methyl-6-(4-hydroxyphenyl)-2-heptene-4-one, and 4-methylene-5-hydroxybisabola-2, 10-diene-9-one which have been shown to inhibit LPS-induced inflammatory responses *in vitro* [116, 117]. Further studies are required to determine whether these constituents may be the lead compound for further development to treat inflammatory diseases including sepsis. It is also worth noting that since curcumin is recognized as the most pharmacologically active constituent isolated from *C. longa*, many studies have also been conducted to improve the anti-inflammatory activity of curcumin through structural modifications [118–123].

### 6.4. *Piper nigrum*

*Piper nigrum* (*P. nigrum*), also widely known as black pepper, is one of the most commonly used spices for culinary purpose [124]. *P. nigrum* originated from India, and it is widely cultivated in Southeast Asia, especially Indonesia and Malaysia. In traditional Chinese medicine, *P. nigrum* has been used for the treatment of cold due to its “warming” properties. On the contrary, *P. nigrum* plays an important role in Ayurvedic medicine as it can improve digestion and treat cold, cough, and fever [124]. Studies have also shown that it possesses antimicrobial, antioxidant, anticancer, and hepatoprotective properties [125–128].

A previous study demonstrated that the ethanol extract of *P. nigrum* was capable of down-regulating NO production in LPS-stimulated macrophages, indicating its inhibitory effect against LPS-mediated inflammatory responses [65]. Although evidence on the therapeutic potential of *P. nigrum* extract against sepsis is lacking, there are several studies that demonstrate the therapeutic potential of two of its active compounds, namely, piperine and pellitorine.

Piperine is the core constituent that gives black pepper its natural aromatic fragrance [129]. Piperine injection via intraperitoneal route improved the survival of mice with endotoxemia induced by LPS, as well as reduced serum levels of TNF-*α* [68]. Another study showed that intragastric administration of piperine reduced the secretion of IL-1*β* in mice intraperitoneally infected with *Escherichia coli*, suggesting its ability to suppress systemic inflammation caused by bacteria [66]. This finding is consistent with results from an *in vitro* study, whereby piperine also suppressed the release of IL-1*β* and HMGB-1 by LPS-stimulated macrophages [66]. Additionally, intraperitoneal injection of piperine also attenuated LPS-induced ALI in mice, most probably due to the reduced production of proinflammatory cytokines such as IL-1*β*, IL-6, and TNF-*α* [69].

Pellitorine is another compound in *P. nigrum* that has garnered much research attention. This compound has been shown to be effective against sepsis in both *in vitro* and *in vivo* models. Pellitorine demonstrated excellent protective effects against vascular barrier dysfunction by reducing permeability, CAMs expression, and leukocytes transmigration in HMGB-1-induced HUVECs and mice [67]. Most importantly, an intravenous dose of pellitorine as low as 9 *μ*g/mouse reduced the mortality of septic mice [67]. However, these evidences suggest that *P. nigrum* may possess antisepsis effect.

Besides piperine and pellitorine, a few studies have also identified other active compounds isolated from *P. nigrum.* Although some of these compounds showed more potent anti-inflammatory activity *in vitro*, their activity in sepsis animal models has not yet been reported. For example, Ngo et al. [130] reported that there are five alkaloids including pellitorine that significantly inhibited NO production in LPS-induced macrophages but chabamide was the most potent among them. Another study reported that some new amide alkaloids isolated from the ethanol extract of *P. nigrum* significantly inhibited LPS-induced NO, IL-1*β*, IL-6, and TNF-*α* production by macrophages [131]. Amongst the isolated amide alkaloids, pipernigramides E-G were shown to exert anti-inflammatory activity in the carrageenan-induced paw edema test [131].

### 6.5. *Syzygium aromaticum/Eugenia caryophyllata*

*Syzygium aromaticum* (*S. aromaticum*) (synonym: *Eugenia caryophyllata*), commonly called cloves, has been used as a spice for centuries as it exudes a distinct aroma due to the presence of its constituent—eugenol. Cloves specifically refer to the nail-shaped dried flower buds from the *S. aromaticum* plant, which originated from east Indonesia [132]. The medicinal properties of cloves include antimicrobial, antinociceptive, antioxidant, and anti-inflammatory effects [133–135].

Although *S. aromaticum* flower buds aqueous extract has been shown to inhibit LPS-induced lung inflammation *in vivo* [73], neither the extract nor the compound isolated from it has been extensively studied for antisepsis properties. Different extracts (acetone, ethanol, and methanol extracts) of *S. aromaticum* flower buds demonstrated antibacterial activity against neonatal sepsis-causing bacteria such as *E. coli*, *Staphylococcus aureus*, *Enterococcus* sp., *Klebsiella* sp., and *Pseudomonas* sp., with the most effective being methanol extract which gave the lowest minimum inhibitory concentration (MIC) [136]. However, this result should be interpreted with caution in terms of its antisepsis potential in general as infections by many other organisms could also lead to sepsis or septic shock. The anti-inflammatory activity of *S. aromaticum* is supported by several studies. For example, the methanol extract of *S. aromaticum* has been shown to inhibit the production of IL-1*β*, IL-6, IL-10, and PGE_2_ in LPS-induced macrophages [70, 71]. Bachiega et al. [71] also demonstrated that eugenol inhibited the production of IL-6 and IL-10 but had no effect on IL-1*β*. Besides eugenol, another compound isolated from the flower buds of *S. aromaticum* called biflorin suppressed LPS-stimulated release of inflammatory mediators such as NO, PGE_2_, TNF-*α*, and IL-6 by macrophages. In the same study, biflorin also improved the survival rate of LPS-induced endotoxemic mice [72]. Overall, the evidence on the antisepsis effect of *S. aromaticum* is still limited, and more extensive studies are required in the future.

### 6.6. *Momordica charantia*

Bitter gourd, or bitter melon, scientifically known as *Momordica charantia* (*M. charantia*), is a well-known vegetable for its bitter taste. The plant is natively found in Asia, Africa, Amazon, and the Caribbean and is now widely cultivated in China and India for commercial purposes. Bitter gourd is valuable as a traditional herbal remedy with proven anti-inflammatory, antioxidant, anticarcinogenic, antimicrobial, and antidiabetic properties [137–140]. After several years of research, *M. charantia* is believed to be one of the most promising natural therapies that could be used to treat diabetes mellitus—a disease that affects numerous people all around the world [141].

Although it is more renowned for its hypoglycemic effect to treat diabetes, increasing evidence supports the antisepsis potential of bitter gourd. An *in vivo* study involving an LPS-stimulated mouse model of sepsis reported reduced levels of inflammatory mediators (TNF-*α*, IL-1*β*, and IL-6) and an elevated level of the anti-inflammatory cytokine IL-10 in septic mice fed with lyophilized powder of wild bitter gourd-supplemented diet [78]. These anti-inflammatory effects correlated with reduced levels of ALT, AST, and C-RP, all of which are markers of liver injury, suggesting its hepatoprotective effect [78]. Several *in vitro* studies have also demonstrated the anti-inflammatory activity of bitter gourd. For example, an *in vitro* study reported that wild bitter gourd fruit extracts (hot water, ethanol, and ethyl acetate extracts) were effective against LPS-induced inflammatory responses in macrophages, mainly by attenuating the secretion of NO and PGE_2_ and the expression of iNOS and pro-IL-1*β* [74]. Its inhibitory effect on NO and PGE_2_ is further supported by other studies which reported similar finding using different extracts of bitter gourd on LPS-stimulated macrophages [75–77]. Together, these findings highlight its potential to prevent the inflammatory response and ensuing organ damage in sepsis.

The active constituent(s) responsible for the antisepsis effects of bitter gourd remains unknown; however, numerous active compounds isolated from *M. charantia* have recently been shown to have *in vitro* anti-inflammatory activity. Notably, seven compounds isolated from the acetone and methanol extracts of bitter gourd inhibited LPS-induced inflammatory responses in macrophages and gentisic acid, 5-O-*β*-d-xyloside, which was isolated for the first time from bitter gourd, has been shown to inhibit the expression of COX-2 and IL-6 [142]. In another study by Shivanagoudra et al. [143], charantoside XI exhibited the most significant anti-inflammatory activity on LPS-induced macrophages compared to the other three cucurbitane-type compounds. The antisepsis potential of these active compounds remains to be explored in future studies.

### 6.7. *Centella asiatica*

In Malaysia, *Centella asiatica* (*C. asiatica*) is commonly known as “*pegaga*” [144]. *C. asiatica* has been traditionally used, especially in India and China, for medicinal purposes including for wound healing, stimulation of neurons, and to treat skin diseases [145]. Numerous pharmacological activities of the plant have been reviewed, including wound healing, antitumor, memory enhancing, antioxidant, anti-inflammatory, and protective effects on the liver, heart, and brain [146].

*C. asiatica* ethanol extract has been shown to have prominent anti-inflammatory effects both *in vitro* and *in vivo*, as indicated by its inhibition of TNF-*α* and PGE_2_ production in LPS-induced macrophages and LPS-induced mice, respectively [79]. While there is limited evidence on the antisepsis potential of *C. asiatica* extract, a few compounds isolated from the plant, namely, asiaticoside, asiatic acid, and madecassoside, have shown antisepsis activities. Intraperitoneal injection of asiaticoside, a triterpenoid saponin, in CLP-induced mice, reduced the serum levels of IL-6 and TNF-*α*, the expression of COX-2 and iNOS in lung tissue, and the severity of lung injury due to sepsis [81]. Importantly, asiaticoside treatment also improved the survival of CLP-induced mice [81]. In a mouse model of LPS-induced ALI, asiaticoside demonstrated similar beneficial effects by decreasing the degree of lung damage [82]. Asiaticoside has also been reported to attenuate liver injury in LPS-/D-galactosamine-induced mice and LPS-induced rats [83, 84]. Asiatic acid is a major triterpene isolated from *C. asiatica*. Injection of asiatic acid via the intranasal route attenuated ALI via downregulation of TLR4 expression, suppression of NF-*κ*B activation, and subsequent inhibition of proinflammatory cytokines (IL-1*β*, IL-6, and TNF-*α*) production in LPS-induced mice [85]. Besides that, a more recent study found that the oral administration of asiatic acid resulted in increased survival rate and reduced organ damage in LPS-induced endotoxemic mice [80]. Madecassoside, another major triterpenoid, has also been associated with tissue protective effects *in vivo*. An intragastric injection of madecassoside has been shown to have cardioprotective effects against myocardial dysfunction in LPS-treated rats by reducing plasma TNF-*α*, preventing the fall in blood pressure and attenuating the severity of tachycardia [86]. Furthermore, an oral administration of madecassoside was able to prevent acute liver failure in LPS-/D-galactosamine-induced mice [87].

**Figure 1:**
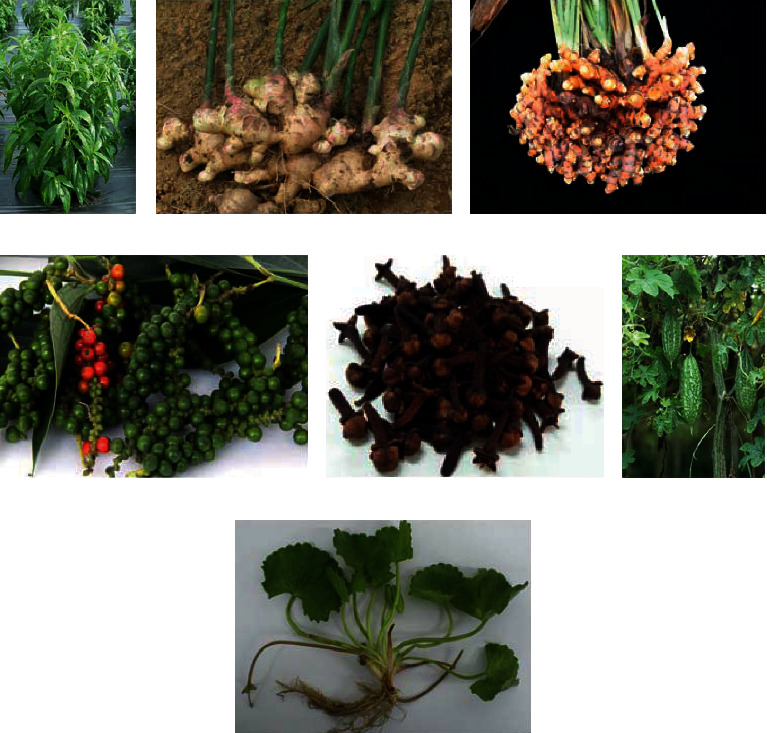
Photos of Malaysian herbal plants with therapeutic potential for sepsis: (a) whole plant of *Andrographis paniculata*; (b) rhizome of *Zingiber officinale*; (c) rhizome of *Curcuma longa*; (d) *Piper nigrum* berries; (e) dried flower buds of *Syzygium aromaticum*; (f) fruits of *Momordica charantia*; and (g) whole plant of *Centella asiatica*. GLOB*in*MED, https://www.globinmed.com/index.php?option=com_content&view=category&id=209&Itemid=143 (accessed 4 March 2020).

Two other triterpenoids, namely, madecassic acid and asiaticoside G, have been shown to have anti-inflammatory effect *in vitro*. Won et al. [147] reported that madecassic acid had a stronger inhibitory effect on the production of NO, PGE_2_, TNF-*α*, IL-1*β*, and IL-6 in LPS-induced macrophages compared to madecassoside. In addition, asiaticoside G, a new ursane-type triterpenoid glycoside isolated from *C. asiatica* leaves, has been shown to have stronger inhibitory effect against LPS-induced NO and TNF-*α* production in macrophages compared to asiaticoside and asiatic acid [148]. Thus, it would be interesting to further investigate whether these two compounds have therapeutic effects in sepsis animal models (see Figure 1).

## 7. Discussion

Sepsis is a major cause of death and morbidity worldwide [8, 12]. The current approaches in managing sepsis patients include initial resuscitation, early diagnosis to allow prompt treatment using specific antibiotics, and identification of infection source and control measures [2]. These have improved the outcomes of sepsis patients; however, the mortality rate remains unreasonably high [4]. Furthermore, the reliance on antibiotics may exacerbate the global threat of multidrug-resistant organisms, leaving no treatment option for sepsis [149]. An alternative approach—a specific antisepsis treatment, is urgently needed to ease the global burden of sepsis due to the tremendous costs incurred in the management of patients [8]. Hence, this review aims to give some insights into Malaysian herbal plants and their active constituents which have the potential to be further developed into alternative treatments for sepsis.

Among the herbal plants discussed in this review, *Z. officinale* and *A. paniculata* are natively found in Malaysia, whilst others are naturalized species since years ago. Based on the literature, *Z. officinale*, or ginger, seems to show the highest potential to be developed for therapeutic use in sepsis. This is evidenced by promising results from different experimental models, both *in vitro* and *in vivo*, which involved different forms of ginger—alcoholic extract of ginger, dried ginger water extract, 6-gingerol, and zingerone [48–55]. The greatest effect was demonstrated by zingerone, a phenoline alkanone isolated from ginger. Zingerone was able to enhance the survival rate and attenuate injury in organs commonly affected by sepsis, which are the kidney, liver, and lung, at a low dose of 0.72 mg/kg in CLP-induced mice [50, 51]. Furthermore, there is an ongoing clinical trial in China that evaluates the efficacy of “*Si-Ni-Tang,*” a traditional Chinese medicine formulation comprising of ginger, in treating sepsis [150]. This further supports the potential use of ginger for sepsis. Other than *Z. officinale*, this traditional remedy is composed of processed *Glycyrrhiza uralensis* and *Aconitum carmichaeli*, based on the rationale that the simultaneous use of herbal plants with different pharmacological activities is likely to be more effective as a treatment for septic shock patients [150]. This is also one of the traditional remedies used to treat sepsis or septic shock as documented in the ancient medical collection called “*Shanghan Lun*” [150].

For some herbal plants, evidence on their antisepsis potential is largely demonstrated by specific constituents or compounds isolated from the plant extracts. For instance, andrographolide is a major constituent in the ethyl acetate fraction of *A. paniculata* [93] and is thought to largely contribute to the antisepsis effect of *A. paniculata* ethyl acetate extract in LPS-induced endotoxemia [44]. Andrographolide at a dose of 1 to 10 mg/kg via intraperitoneal injection was demonstrated to attenuate acute lung injury and acute liver injury in LPS-induced and LPS-/D-galactosamine-induced mice, respectively [46, 47], whereas a relatively lower dose (3.5 to 7 *μ*g/mouse) via intravenous injection enhanced the survival of CLP-induced mice [45]. For *C. longa*, curcumin is the major constituent and it has been shown to promote survival and exert organ-protective effects against sepsis-induced injury in numerous studies [56–64, 106–114]. It is likely that the antisepsis effect of *C. longa*, as indicated by the enhanced survival rate of LPS-induced mice receiving CLEN treatment [56], is attributed to curcumin. Both andrographolide and curcumin are the most pharmacologically active constituent and the most widely studied phytochemical in their respective plants [89, 151].

In comparison to *A. paniculata* and *C. longa*, more than one active constituent with antisepsis potential has been identified from *P. nigrum* and *C. asiatica*. Specifically, these constituents are piperine and pellitorine from *P. nigrum* and asiaticoside and asiatic acid and madecassoside from *C. asiatica*. Piperine was demonstrated to be effective in inhibiting the systemic inflammatory responses induced by LPS as well as *E. coli* [66, 68, 69], whereas pellitorine is more likely to be an active compound that protects the endothelial barrier as it has been shown to inhibit vascular endothelial dysfunction, both *in vitro* and *in vivo* [67]. Piperine and pellitorine can also be found in the other member within the same genus such as *P. longum*, which is commonly known as long pepper [152]. Asiaticoside, asiatic acid, and madecassoside, on the contrary, were demonstrated to protect against organ dysfunction [80–87]. These three active principles, together with madecassic acid, are triterpenes that are believed to be the major components that contribute to the medicinal value of *C. asiatica* [144]. Notably, these active constituents were all given within a similar dose range (1 to 100 mg/kg) via different routes (oral, intragastric, intraperitoneal, and intranasal), except pellitorine which was injected intravenously at a substantially lower dose of 4.5 to 9.0 *μ*g/mouse [67]. This finding indicates that the active compounds may be required at a relatively lower dose to achieve the desired therapeutic effects if they are administered intravenously compared to the other routes.

Among the plants discussed in this review, there are relatively fewer studies on *S. aromaticum* and *M. charantia*. The aqueous extract of *S. aromaticum* flower buds has been shown to inhibit lung inflammation in LPS-induced mice [73], but the active constituent responsible for the anti-inflammatory activity *in vivo* remains unknown. Biflorin, an active compound isolated from the butanol-soluble fraction of the ethanol extract of *S. aromaticum* flower buds, however, has been shown to improve the survival rate of LPS-induced endotoxemic mice [72]. Thus, it is likely that there is other active constituent present in the flower buds of *S. aromaticum* that may also have antisepsis potential. Unlike the other active constituents mentioned, biflorin was originally isolated from *Capraria biflora*, a perennial shrub distributed in North and South America [153]. In contrast to the other plants, *M. charantia* has the least evidence to support its antisepsis potential. In particular, the lyophilized powder of *M. charantia*, given in the form of supplemented food pellets, has been shown to have *in vivo* anti-inflammatory and hepatoprotective effects in LPS-induced mice [81]. Although this may imply that *M. charantia* may treat sepsis by suppressing the inflammatory response and associated liver damage, further studies using different models of sepsis are required. Furthermore, the active compound responsible for the therapeutic effects is yet to be identified.

In summary, the active constituents which are predominantly found in the Malaysian herbal plants have shown great antisepsis effects in the preclinical studies and should be further evaluated for their therapeutic potential against sepsis in the clinical settings.

## 8. Conclusion

The lack of a cure for sepsis and the sole dependence on antibiotics for sepsis management highlight the need to consider alternative treatments for sepsis. Some herbal plants available in Malaysia, particularly their active constituents, have shown promising antisepsis potential and are worth to be tested clinically in order to find an effective treatment for sepsis.

## Figures and Tables

**Table 1:** Malaysian herbal plants and their active constituents with potential therapeutic applications in sepsis.

Scientific name (local name)	Family	Experimental model	Type of sample (concentration or dose used)/mode and duration of treatment	Antisepsis properties	Positive drug control	References
*Andrographis paniculata* (hempedu bumi)	Acanthaceae	*In vitro*	Ethyl acetate extract (2–10 *μ*g/mL), pretreatment (1 hr)	(i) Inhibits NO and PGE_2_ production (ii) Inhibits NF-*κ*B transcriptional activity in LPS-/IFN-*γ*-induced macrophages	Helenalin, NF-*κ*B inhibitor (10 *μ*M), pretreatment (1 hr)	[43]
			Ethyl acetate extract (2.5–20 *μ*g/mL), pretreatment (1 hr)	(i) Decreases NO, MIP-2, TNF-*α*, and IL-6 production in LPS-/IFN-*γ*-induced macrophages	Helenalin, NF-*κ*B inhibitor (10 *μ*M), pretreatment (1 hr)	[44]
			Andrographolide (1–10 *μ*M), posttreatment (6 hr)	(i) Reduces endothelial permeability (ii) Inhibits CAMs expression (iii) Suppresses monocyte adhesion and transmigration (iv) Inhibits TNF-*α* and IL-1*β* production in HMGB-1-induced HUVECs	Emodin-6-O-*β*-D-glucoside (EG) (10 *μ*M), posttreatment (6 hr)	[45]
		*In vivo*	Ethyl acetate extract (0.78–3.12 mg/kg) via oral administration, daily for one week before LPS challenge	(i) Improves survival rate (ii) Reduces serum levels of TNF-*α*, MIP-2, and NO in LPS-induced mice	Pyrrolidine dithiocarbamate (PDTC), NF-*κ*B inhibitor (50 mg/kg) via intraperitoneal injection, 1 hr before LPS stimulation	[44]
			Andrographolide (3.5–7.0 *μ*g/mouse) via intravenous injection 16 hr after the CLP procedure	(i) Inhibits HMGB-1 production (ii) Decreases vascular permeability (iii) Suppresses leukocytes and neutrophils migration (iv) Improves survival rate in CLP-induced mice	Emodin-6-O-*β*-D-glucoside (EG) (9.0 *μ*g/mouse) via intravenous injection 16 hr after the CLP procedure	[45]
			Andrographolide (1–10 mg/kg) via intraperitoneal injection 10 min after LPS challenge	(i) Attenuates ALI (ii) Reduces TNF-*α*, IL-1*β*, and IL-6 levels in BALF (iii) Inhibits NF-*κ*B activation and its DNA binding activity in LPS-induced mice	NA	[46]
			Andrographolide (2.5–10 mg/kg) via intraperitoneal injection 1 hr after LPS/D-galactosamine challenge	(i) Attenuates acute liver injury (ii) Reduces serum levels of ALT and AST (iii) Inhibits hepatic TNF-*α* and IL-1*β* production (iv) Inhibits NF-*κ*B activation in LPS-/D-galactosamine-induced mice	NA	[47]
*Zingiber officinale* (halia)	Zingiberaceae	*In vitro*	Alcoholic ginger extract (1 *μ*l/mL), cotreatment (24 hr)	(i) Inhibits production of proinflammatory cytokines (IL-1*β*, IL-12, and TNF-*α*) (ii) Inhibits production of chemokines (RANTES and MCP-1) in LPS-induced macrophages	NA	[48]
			6-Gingerol (1 *μ*g/mL), cotreatment (24 hr)	(i) Inhibits production of proinflammatory cytokines (IL-1*β*, IL-12, and TNF-*α*) (ii) Inhibits production of chemokine (RANTES) in LPS-induced macrophages	NA	[49]
			Zingerone (10–50 *μ*M), posttreatment (6 hr)	(i) Inhibits endothelial hyperpermeability (ii) Suppresses expression of CAMs (ICAM and VCAM) (iii) Reduces adhesion and migration of neutrophils in HMGB-1-induced HUVECs	NA	[50]
			Zingerone (10–50 *μ*M), posttreatment (6 hr)	(i) Inhibits endothelial hyperpermeability (ii) Suppresses expression of CAMs (ICAM and VCAM) (iii) Reduces adhesion and migration of neutrophils in TGFBIp-induced HUVECs	NA	[51]
		*In vivo*	Dried ginger extract dissolved in distilled water (100–1000 mg/kg) via oral administration for 3 days before LPS challenge	(i) Decreases IL-6 and IFN-*γ* production (ii) Inhibits iNOS and COX-2 expression in LPS-induced mice	NA	[52]
			6-Gingerol (40 mg/kg) via intragastric administration for 5 days before the CLP procedure	(i) Attenuates liver injury (ii) Decreases serum levels of ALT and AST (iii) Reduces serum level of IL-1*β* in CLP-induced mice	NA	[53]
			Zingerone (0.36 and 0.72 mg/kg) via intravenous injection 12 hr and 50 hr after the CLP procedure	(i) Improves survival rate (ii) Alleviates tissue injury (renal, hepatic, and pulmonary injury) in CLP-induced mice	NA	[50]
			Zingerone (0.36 and 0.72 mg/kg) via intravenous injection 12 hr and 50 hr after TGFBIp injection	(i) Improves survival rate (ii) Alleviates tissue injury (renal, hepatic, and pulmonary injury) in TGFBIp-induced CLP mice	NA	[51]
			Zingerone (10–40 mg/kg) via intragastric administration 1 hr before LPS challenge	(i) Attenuates ALI (ii) Reduces serum levels of TNF-*α* and IL-1*β* in LPS-induced mice	Dexamethasone (5 mg/kg) via intragastric administration 1 hr before LPS challenge	[54]
			Zingerone (10–40 mg/kg) via intraperitoneal injection 1 hr after LPS challenge	(i) Decreases serum levels of blood urea nitrogen (BUN) and creatinine (ii) Attenuates kidney injury (iii) Reduces levels of IL-1*β*, IL-6, and TNF-*α* in the serum and kidney tissue in LPS-induced mice	Viral inhibitory peptide for TLR4 (VIPER) (0.1 mg/kg) via intravenous injection 2 hr before LPS challenge	[55]
*Curcuma longa* (kunyit)	Zingiberaceae	*In vitro*	*C. longa* extract-loaded nanoemulsion (CLEN) (5 *μ*g/mL), cotreatment (24 hr)	(i) Inhibits NO production (ii) Suppresses HMGB-1 release (iii) Inhibits iNOS expression in LPS-stimulated macrophages	NA	[56]
		*In vivo*	CLEN (50 mg/kg) via intraperitoneal injection together with LPS challenge	(i) Improves survival rate in LPS-induced mice	NA	[56]
			Curcumin (3.5 *μ*mol/kg) via intravenous injection 5 hr after the CLP procedure	(i) Improves survival rate (ii) Reduces levels of tissue injury markers (ALT, AST, and lactate) (iii) Decreases TNF-*α* level in CLP-induced mice	NA	[57]
			Curcumin (100 mg/kg) via intraperitoneal injection for 48 hr before the CLP procedure	(i) Improves survival rate (ii) Attenuates blood-brain barrier dysfunction (iii) Inhibits leukocytes and platelets adhesion in CLP-induced mice	NA	[58]
			Curcumin (10 mg/kg) via intranasal administration 1 hr before LPS instillation	(i) Attenuates ALI (ii) Decreases NO and TNF-*α* levels (iii) Inhibits vascular leakage in LPS-induced mice	Dexamethasone (5 mg/kg) via intraperitoneal injection 1 hr before LPS instillation	[59]
			Curcumin (200 mg/kg) via intraperitoneal injection 30 min before LPS challenge	(i) Attenuates ALI (ii) Reduces TNF-*α*, IL-6, and MIP-2 levels in BALF in LPS-induced mice	NA	[60]
			Curcumin (20–80 mg/kg) via oral administration daily for 4 weeks before LPS challenge	(i) Inhibits production of proinflammatory cytokines (IL-1*β*, IL-6, and TNF-*α*) (ii) Prevents liver failure in LPS-induced mice	NA	[61]
			Curcumin (20 mg/kg) via intraperitoneal injection 1 hr before LPS challenge	(i) Improves survival rate (ii) Decreases the levels of TNF-*α* and inflammatory cells in BALF in LPS-induced mice	Dexamethasone (1 mg/kg) via intraperitoneal injection 1 hr before LPS challenge	[62]
			Curcumin (200 mg/kg/day) via intragastric administration for 3 days	(i) Attenuates cardiac dysfunction in CLP-induced mice	NA	[63]
			Curcumin (50–100 mg/kg) via intraperitoneal injection 3 hr before or after LPS challenge	(i) Improves vascular function (ii) Maintains normal heart rate (iii) Restores normal arterial blood pressure in LPS-induced mice	NA	[64]
*Piper nigrum* (lada hitam)	Piperaceae	*In vitro*	Ethanol extract (NA)	(i) Inhibits NO production in LPS stimulated macrophages	NA	[65]
			Piperine (20–80 *μ*M), pretreatment (4 hr)	(i) Inhibits IL-1*β* production (ii) Inhibits HMGB-1 production in LPS-stimulated macrophages	NA	[66]
			Pellitorine (3–20 *μ*M), pretreatment (6 hr)	(i) Reduces endothelial permeability (ii) Inhibits CAMs expression (iii) Suppresses leukocytes adhesion and migration in HMGB-1-induced HUVECs	Emodin-6-O-*β*-D-glucoside (EG) (10 *μ*M), pretreatment (6 hr)	[67]
		*In vivo*	Piperine (1 and 5 mg/kg) via intraperitoneal injection 1 hr before LPS challenge	(i) Improves survival rate (ii) Inhibits TNF-*α* production in LPS-induced mice	NA	[68]
			Piperine (20 mg/kg) via intragastric administration for 5 days before *E. coli* injection	(i) Reduces serum and BALF levels of IL-1*β* (ii) Reduces pro-IL-1*β* and IL-1*β* protein levels in colonic tissue in *E. coli*-infected mice	NA	[66]
			Piperine (15–60 mg/kg) via intraperitoneal injection 1 hr after LPS challenge	(i) Attenuates ALI (ii) Reduces IL-1*β*, IL-6, and TNF-*α* levels in BALF (iii) Inhibits NF-*κ*B activation in LPS-induced mice	NA	[69]
			Pellitorine (4.5–9.0 *μ*g/mouse) via intravenous injection 6 hr before HMGB-1 challenge	(i) Improves survival rate (ii) Reduces vascular permeability (iii) Inhibits CAMs expression (iv) Inhibits leukocytes adhesion and migration in HMGB-1-induced mice	Emodin-6-O-*β*-D-glucoside (EG) (9.0 *μ*g/mouse) via intravenous injection 6 hr before HMGB-1 challenge	[67]
*Syzygium aromaticum* (bunga cengkih)	Myrtaceae	*In vitro*	Methanol extract (10 *μ*g/mL), cotreatment (9–24 hr)	(i) Inhibits PGE_2_ production in LPS-induced macrophages	NA	[70]
			Methanol extract (100 *μ*g/well), pretreatment (2 hr) and posttreatment (22 hr)	(i) Inhibits IL-1*β*, IL-6, and IL-10 production in LPS-induced macrophages	Dexamethasone (100 *μ*M)	[71]
			Biflorin (15–60 *μ*M), pretreatment (1 hr)	(i) Inhibits NO, PGE_2_, IL-6, and TNF-*α* production (ii) Inhibits iNOS and COX-2 expression in LPS-stimulated macrophages	L-NIL (20 *μ*M) and NS-398 (3 *μ*M), pretreatment (1 hr)	[72]
		*In vivo*	Aqueous extract (200 mg/kg) via intraperitoneal injection for 2 days before LPS challenge	(i) Inhibits lung inflammation in LPS-induced mice	NA	[73]
			Biflorin (5 and 10 mg/kg) via intraperitoneal injection 1 hr before LPS challenge	(i) Improves survival rate (ii) Reduces TNF-*α* production (iii) Inhibits iNOS and COX-2 expression in LPS-induced mice	NA	[72]
*Momordica charantia* (peria)	Cucurbitaceae	*In vitro*	Hot water (500–2000 *μ*g/mL), ethanol (12.5–50 *μ*g/mL), and ethyl acetate (25–100 *μ*g/mL) extracts, cotreatment (6–24 hr)	(i) Inhibits NO and PGE_2_ production (ii) Inhibits iNOS and pro-IL-1*β* expression in LPS-stimulated macrophages	Gentisic acid (1 mM), cotreatment (18–24 hr)	[74]
			Methanol extract (100–200 *μ*g/mL), pretreatment (30 min)	(i) Inhibits NO and PGE_2_ production (ii) Inhibits iNOS and COX-2 expression in LPS-induced macrophages (i) Inhibits PGE_2_ production in LPS-induced macrophages	L-NAME (1–1.5 mM) and indomethacin (0.5–5 *μ*M), pretreatment (30 min)	[75]
			Water extract (0.01–0.5 mg/mL), cotreatment (12–18 hr)		NA	[76]
			Ethanol, ethyl acetate, petroleum ether, and aqueous extracts (0.1 mg/mL), pretreatment (12 hr)	(i) Inhibits NO production in LPS-stimulated macrophages	Ibuprofen (0.1 mg/mL), pretreatment (12 hr)	[77]
		*In vivo*	Lyophilized powder (1%–10% (w/w)) in food pellets daily for 4 weeks before LPS challenge	(i) Reduces serum levels of proinflammatory cytokines (TNF-*α*, IL-1*β*, and IL-6) and increases serum level of anti-inflammatory cytokine (IL-10) (ii) Inhibits expression of NF-*κ*B, iNOS, and COX-2 (iii) Diminishes liver injury biomarkers (ALT, AST, and C-RP) in LPS-induced mice	Pyrrolidine dithiocarbamic acid ammonium salt (PDTC) (50 mg/kg) via intravenous injection 1 hr before LPS challenge	[78]
*Centella asiatica* (pegaga)	Apiaceae	*In vitro*	Ethanol extract (3.91–1000 *μ*g/mL), posttreatment (24 hr)	(i) Inhibits NO, TNF-*α*, and PGE2 production in LPS-induced macrophages	Dexamethasone (5 *μ*M), posttreatment (24 hr)	[79]
			Asiatic acid (10–40 *μ*M), cotreatment (48 hr)	(i) Inhibits NO, IL-1*β*, and IL-6 production	NA	[80]
				(ii) Inhibits NF-*κ*B activation in LPS-induced macrophages		
		*In vivo*	Ethanol extract (300 and 350 mg/kg) via oral administration after LPS challenge for 11 days	(i) Reduces serum levels of TNF-*α* and PGE2 in LPS-induced mice	Rivastigmine (5 mg/kg) via oral administration after LPS challenge for 11 days	[79]
			Asiaticoside (45 mg/kg) via intraperitoneal injection 1 hr before the CLP procedure	(i) Attenuates ALI (ii) Reduces serum levels of IL-6 and TNF-*α* (iii) Inhibits COX-2 and iNOS expression in lung tissue (iv) Inhibits NF-*κ*B activation in CLP-induced mice	NA	[81]
			Asiaticoside (15–45 mg/kg) via intraperitoneal injection 1 hr before LPS challenge	(i) Attenuates ALI (ii) Reduces IL-6 and TNF-*α* levels in BALF (iii) Inhibits NF-*κ*B activation in LPS-induced mice	NA	[82]
			Asiaticoside (10 and 20 mg/kg) via oral administration for 3 days before the LPS/D-galactosamine challenge	(i) Improves survival rate (ii) Attenuates liver injury (iii) Reduces serum levels of ALT and AST (iv) Decreases TNF-*α* level in serum and hepatic tissue in LPS-/D-galactosamine-induced mice	Silymarin (50 mg/kg) via oral administration for 3 days before LPS/D-galactosamine challenge	[83]
			Asiaticoside (3–30 mg/kg) via oral administration at 24 hr before the LPS challenge and every 8 hr thereafter	(i) Reduces serum levels of IL-1*β*, IL-6, and TNF-*α* (ii) Attenuates liver injury in LPS-treated rats	Indomethacin (10 mg/kg) via oral administration at 24 hr before LPS challenge and every 8 hr thereafter	[84]
			Asiatic acid (25–100 mg/kg) via intranasal administration 1 hr after LPS challenge	(i) Attenuates ALI (ii) Reduces IL-1*β*, IL-6, and TNF-*α* levels in BALF (iii) Inhibits NF-*κ*B activation in LPS-induced mice	NA	[85]
			Asiatic acid (10 and 30 mg/kg) via oral administration for 3 days before LPS challenge	(i) Improves survival rate (ii) Attenuates organ (lung, liver, and kidney) damage (iii) Decreases serum levels of IL-1*β*, IL-6, ALT, and BUN in LPS-induced mice	NA	[80]
			Madecassoside (20 mg/kg) via intragastric administration for 5 days before LPS challenge	(i) Prevents myocardial dysfunction (ii) Delays the drop in blood pressure (iii) Attenuates tachycardia in LPS-treated rats	NA	[86]
			Madecassoside (20 and 40 mg/kg) via oral administration for 10 days before LPS challenge	(i) Prevents acute liver failure (ii) Reduces serum levels of ALT and AST (iii) Inhibits IL-1*β*, IL-6, and TNF-*α* production in liver tissue (iv) Inhibits iNOS and COX-2 expression in LPS-/D-galactosamine-induced mice	Silibinin (100 mg/kg) via oral administration for 10 days before LPS challenge	[87]

## Data Availability

The data used to support the findings in the study are available on reasonable request to the corresponding author.

## Supplementary Materials

Supplementary Materials The list of 64 Malaysian herbal plants documented in the Malaysian Herbal Monograph 2015 is provided in the Supplementary Material (Table A).
