# Supplementary material for: A Review of Malaysian Herbal Plants and Their Active Constituents with Potential Therapeutic Applications in Sepsis
Source: Evid Based Complement Alternat Med. 2020 Oct 28;2020:8257817. doi: 10.1155/2020/8257817 (PMC7641701; doi:10.1155/2020/8257817)
Supplement: Supplementary Materials — The list of 64 Malaysian herbal plants documented in the Malaysian Herbal Monograph 2015 is provided in the Supplementary Material (Table A). [file 8257817.f1.docx]

Table A: List of herbal plants documented in Malaysian Herbal Monograph 2015.

| **No.** | **Scientific Name** | **Common Name** |
| --- | --- | --- |
| 1 | *Acorus calamus* L. | Sweet flag |
| 2 | *Alpinia conchigera* Griffith | Lesser alpinia |
| 3 | *Andrographis paniculata* (Burm.f.) Nees | King of bitters |
| 4 | *Areca catechu* L. | Betelnut palm |
| 5 | *Ardisia elliptica* Thunb. | Shoebutton ardisia |
| 6 | *Blumea balsamifera* (L.) DC. | *‘Sembong’ (Malay)* |
| 7 | *Brucea javanica* (L.) Merill | Java brucea |
| 8 | *Centella asiatica* (L.) Urban | Asiatic Pennywort |
| 9 | *Chromolaena odorata* R.M.King & H.Rob | Siam weed |
| 10 | *Cinnamomum iners* Reinw. ex. Blume | Wild cinnamon |
| 11 | *Cinnamomum verum* J.Presl | Ceylon cinnamon |
| 12 | *Citrus aurantiifolia* (Christm.) Swingle | Key lime |
| 13 | *Citrus microcarpa* Bunge | Calamansi |
| 14 | *Clinacanthus nutans* (Burm.f.) Lindau | *‘Belalai gajah’ (Malay)* |
| 15 | *Cosmos caudatus* Kunth | ‘*Ulam raja’ (Malay)* |
| 16 | *Curcuma longa* L. | Turmeric |
| 17 | *Curcuma zanthorrhiza.* Roxb. | Jawa ginger |
| 18 | *Cymbopogon citratus* (DC.) Stapf. | Lemon grass |
| 19 | *Cymbopogon nardus* (L.) Rendle | Citronella grass |
| 20 | *Elaeis guineensis* Jacq. | African oil palm |
| 21 | *Elephantopus scaber* L. | Elephant’s foot |
| 22 | *Eugenia caryophyllata* Thunb. | Clove |
| 23 | *Eurycoma longifolia* Jack | *‘Tongkat Ali’ (Malay)* |
| 24 | *Fibraurea tinctoria* Lour. | *‘Akar badi’ (Malay)* |
| 25 | *Ficus deltoidea* Jack | Mistletoe fig |
| 26 | *Gendarussa vulgaris* Nees. | Willow-leaved Justicia |
| 27 | *Goniothalamus macrophyllus* (Blume) Hook. f. & Thomson. | ‘*Selada’ (Malay)* |
| 28 | *Goniothalamus scortechinii* King | *‘Akar gajah beranak’ (Malay)* |
| 29 | *Gynura procumbens* (Lour.) Merr. | Longevity spinach |
| 30 | *Hibiscus sabdariffa* Linn | Roselle |
| 31 | *Illicium verum.* Hook.f. | Star anise |
| 32 | *Kaempferia galanga* Linn. | Sand ginger |
| 33 | *Labisia pothoina* Lindl. | *‘Kacip fatimah’ (Malay)* |
| 34 | *Labisia pumila* (Blume) Fern. | *‘Kacip fatimah’ (Malay)* |
| 35 | *Languas galanga* (L.) Stuntz | Greater galangal |
| 36 | *Melastoma malabathricum* L. | *‘Senduduk’ (Malay)* |
| 37 | *Mitragyna speciosa* (Korth.) Havil. | Kratom |
| 38 | *Momordica charantia* L. | Bitter melon |
| 39 | *Morinda citrifolia* L. | Indian Mulberry |
| 40 | *Moringa oleifera* Lam. | Horseradish tree |
| 41 | *Myristica fragrans* Houtt | Nutmeg |
| 42 | *Ocimum basilicum* L. | Sweet basil |
| 43 | *Ocimum tenuiflorum* L. | Holy basil |
| 44 | *Oroxylum indicum* (L.) Kurz | Broken bones tree |
| 45 | *Orthosiphon aristatus* (Blume) Miq. | Cat’s whiskers |
| 46 | *Persicaria minor* (Huds.) Opiz | Small water pepper |
| 47 | *Phyllagathis rotundifolia* (Jack) Blume | Solomon’s sole |
| 48 | *Phyllanthus niruri* L. | Gale of the wind |
| 49 | *Piper betle* L. | Betel pepper |
| 50 | *Piper nigrum* L. | Black pepper |
| 51 | *Piper sarmentosum* Roxb. | Wild pepper |
| 52 | *Polyalthia bullata* King | *‘Tongkat Ali hitam’ (Malay)* |
| 53 | *Prismatomeris tetrandra* (Roxburgh) K. Schum. | Robin’s coffee |
| 54 | *Rafflesia hasseltii* Suringar | NA |
| 55 | *Senna alata.* (L.) Roxb. | Candle bush |
| 56 | *Smilax myosotiflora* A. DC. | *‘Ubi jaga’ (Malay)* |
| 57 | *Stemona tuberosa* Lour. | *‘Galak tua’ (Malay)* |
| 58 | *Tinospora crispa* (L.) Hook. f. & Thomson | *‘Patawali’ (Malay)* |
| 59 | *Trigonella foenum-graecum* L. | Fenugreek |
| 60 | *Vitex negundo* L. | Chinese chaste tree |
| 61 | *Zingiber cassumunar* Roxb. | Cassumunar ginger |
| 62 | *Zingiber officinale* Rosc. | Ginger |
| 63 | *Zingiber ottensii* Valeton | Borneo red beehive ginger |
| 64 | *Zingiber zerumbet* (L.) Roscoe ex Sm. | Shampoo ginger |
